# An Extended Production and Inspection Model with Nonrigid Demand

**DOI:** 10.1155/2013/623075

**Published:** 2013-12-12

**Authors:** Neng-Hui Shih, Chih-Hsiung Wang

**Affiliations:** Department of Business Administration, National Pingtung Institute of Commerce, 51 Min-Sheng E. Road, Pingtung 900, Taiwan

## Abstract

This paper extends a previous production and inspection (PI) model in relation to market demand that is nonrigid to consider an imperfect process that has a general hazard rate instead of a constant failure rate. Products are produced by an imperfect process that may shift randomly from the IN state to the OUT state. When the process is in the OUT state, it has a higher probability of producing a nonconforming product than when it is in the IN state. To achieve the zero defects policy, all products before delivery to the market should be inspected and the inspection order follows their production sequence. Furthermore, the inspection information from all previous products is used to decide either that the next candidate product should be inspected or that the inspection procedure for the current production lot should be terminated. When the inspection procedure is terminated, the remaining unmet demand is regarded as a shortage. An algorithm is developed to determine an optimal PI policy that minimizes the expected total cost, which includes the cost of inspection, of shortage, and of production.

## 1. Introduction 

In practice the quality status of production systems is never perfect due to corrosion, aging, and other factors; these result in a system that may randomly shift from the in-control state to the out-of-control state where the latter starts to produce more nonconforming products (e.g., see Wang [1]). Owing to the fact that nonconforming products are inevitable from an imperfect production system, the traditional economic production quantity (EPQ; e.g., refer to Stevenson [2]) needs to be amended. For example, both Porteus [3] and Rosenblatt and Lee [4] suggest using a smaller EPQ than the classical EPQ in order to diminish the number of nonconforming products in order that the cost of process setup, of inventory holding, and of defective products can be economically balanced.

Online inspection-maintenance (IM) is an effective way of reducing the number of nonconforming products in a production batch. This is because, when the process has shifted to the out-of-control state from the in-control state, the online IM helps to detect the out-of-control situation. This then allows recovery of the system into the in-control state, which shortens the duration the system remains in the out-of-control state. Nevertheless, frequent IM is always expensive and can result in huge costs. By balancing the cost of the process IM, of setup, of inventory holding, and of the nonconforming products in a production cycle, Tseng [5] (also refer to Wang [6]) explored the optimal IM schedule for a given production run length under the condition where the IM intervals are equal. Since a control chart is often used to monitor the quality status of an imperfect production system in practice, Rahim [7] studied how to jointly determine the optimal production batch size and the design of a control chart to monitor the process.

In a manner that is different from the methodology of using online IM to control process quality in order to reduce the number of nonconforming items in a production lot, it is possible to perform product quality control after the whole production batch is complete, called “off-line quality control” (e.g., see Raz et al. [8]). Using off-line quality control often happens when the system cannot be interrupted to maintain the system while the process is in operations or when such an interruption will incur a significant cost (see Rosenblatt and Lee [4]). The food industry (see Raz et al. [8]) and PCB manufacturing (see Sheu et al. [9]) are practical examples of using off-line quality control. Raz et al. [8] developed an economical off-line inspection and disposition model for such systems in order to balance the costs between inspection, the penalty for wrongly accepting a nonconforming product, and the penalty for wrongly rejecting a conforming product. Later, Raz et al.'s [8] model was reformulated by Sheu et al. [9] in order to consider two possible types of inspection errors.

A zero defects policy has been advocated for industrial production since the 1960s because nonconforming products will always result in companies losing their reputation and/or cause an upsurge in defective costs as a result of the cost of returns or warranty costs. When off-line quality control is considered, the zero defects policy can be easily achieved using Raz et al.'s [8] model by setting both cost parameter values for wrongly accepting a nonconforming product and rejecting a conforming product as infinity. However, when the process is subject to manufacturing variations, Raz et al.'s [8] model becomes invalid. To meet the zero defects policy when the process is subject to manufacturing variations (namely, a ZDMV problem), it is necessary to inspect each product before delivering it to the market. To deal with the ZDMV problem, Anily and Grosfeld [10] develop a production and inspection (PI) model for market demand that is nonrigid.

The purpose of this paper is to extend the PI model proposed by Anily and Grosfeld [10] to consider the process when there is an arbitrary discrete shift distribution instead of a constant hazard rate. The sections are arranged as follows. In Section 2, the imperfect process is described. In Section 3, an algorithm for finding an optimal PI policy is developed. Finally, concluding remarks are made in Section 4.

## 2. The Imperfect Process 

Assume that a production batch is produced from an imperfect system with the capacity *n*_*U*_ < *∞*. Here, we adopt the imperfect production system described in, for example, Wang and Sheu [11]. When we start to produce a batch, the system is set up and maintained thus is as good as new and in the in-control (IN) state; these actions have the cost *α*. The varied cost for producing a product is *β*. While producing, the imperfect system may shift at random from the IN state to the out-of-control (OUT) state. Let the random variable *M* represent the number of produced products when the process shifts from the IN state to the OUT state since the last set up of the process. The probability that the first *j* products are manufactured in the IN state is denoted by $\text{Pr}\left(M>j\right)={\bar{P}}_{j}$ with $1={\bar{P}}_{0}\geq {\bar{P}}_{1}\geq {\bar{P}}_{2}\geq \cdots$. In addition, ${\bar{P}}_{j-1}-{\bar{P}}_{j}$ is denoted by *p*_*j*_ for *j* = 1,2,…. The property of the manufacturing variations of the process also considered, that is the probability of producing a conforming product given that when the system is in the IN (the OUT state) is *θ*_0_ (*θ*_1_), where 0 < *θ*_1_ < *θ*_0_ < 1.

When a production batch is completed from the imperfect production system, an inspection policy is used for economically controlling product quality where the inspection is assumed to be perfect. As in Anily and Grosfeld [10], the principle that the first produced product is first inspected (FPFI) is adopted. Performing inspections according to the FPFI strategy is able to economically produce more conforming products (also see Anily and Grosfeld [10]) since the quality status of the system producing the initial products in a batch is better than the status of producing the tailed products in a batch.

Let **Y**_*j*_ be the quality status of the first *j* inspected products in a batch. The outcome of **Y**_*j*_ is denoted by **y**_*j*_ = (*y*_1_, *y*_2_,…, *y*_*i*_,…, *y*_*j*−1_, *y*_*j*_), where *y*_*i*_ = 0 or 1, which represents the *i*th product in the batch that has been inspected to be conforming or nonconforming, respectively, for *i* = 1,2,…. Under the FPFI strategy, when the first *j* products have been inspected, one can use all of their inspection information to infer the probability (denoted by *x*_*j*+1_^**y**_*j*_^) that the process is in the IN state while the *j* + 1th product is completely produced, where

(1) xj+1yj=P¯j+1∏i=1j[(1−yi)θ0+yi(1−θ0)]×(∑i=1j{∏k=1i−1[(1−yk)θ0+yk(1−θ0)]×∏k=ij[(1−yk)θ1+yk(1−θ1)]}pi+∏i=1j[(1−yi)θ0+yi(1−θ0)]P¯j)−1,

for *j* = 1,2,…, and $x_{1}^{y_{0}}={\bar{P}}_{1}$. See the Appendix for a derivation of *x*_*j*+1_^**y**_*j*_^.

When the process does not have manufacturing variations, that is, *θ*_0_ = 1 and *θ*_1_ = 0, one has

(2) $$\begin{array}{c} x_{j+1}^{y_{j}}=\frac{\prod_{i=1}^{j}\left(1-y_{i}\right){\bar{P}}_{j+1}}{\sum_{i=1}^{j}\left\{\prod_{k=1}^{i-1}\left(1-y_{k}\right)\prod_{k=i}^{j}y_{k}\right\}p_{i}+\prod_{i=1}^{j}\left(1-y_{i}\right){\bar{P}}_{j}}. \end{array}$$

In this case, the first nonconforming unit is the point at which the process shifts from the IN state into the OUT state. For example, if the 2nd unit is the first nonconforming unit, then one has **y**_2_ = (*y*_1_ = 0, *y*_2_ = 1), which implies that

(3) $$\begin{array}{c} x_{3}^{y_{2}}=\frac{\prod_{i=1}^{2}\left(1-y_{i}\right){\bar{P}}_{3}}{\left[\prod_{k=1}^{2}y_{k}p_{1}+\left(1-y_{1}\right)y_{2}p_{2}\right]+\prod_{i=1}^{2}\left(1-y_{i}\right){\bar{P}}_{2}}=0. \end{array}$$

The last equation shows that the 3rd product must be produced in the OUT state.

## 3. The PI Problem with Nonrigid Demand 

Consider facing a nonrigid demand *D* ≥ 1 and a single production batch with a size of *N* that is used to satisfy the demand, where *N* ≤ *n*_*U*_. Under the FPFI strategy, if there are uninspected products left, then one can choose to inspect the next uninspected product or terminate the inspection procedure. The unit inspection cost is *γ* ($/product). When a product is inspected to be conforming, it can then be used to satisfy the demand; otherwise, the product is discarded. If one decides to terminate the inspection procedure even if there are uninspected products left, then the remaining unmet demand (RUD) will incur a unit shortage cost, *s* ($/product), where *γ* < *s* (see Anily and Grosfeld [10]). This reveals that, through inspection, it is possible to obtain conforming products that are able to satisfy the RUD, which helps reduce the total shortage cost. The objective of this section is to find the optimal production lot size, *N*_*D*_, which minimizes the expected total cost, including the cost of inspection, of shortage, and of production under the FPFI strategy.

Suppose the first *j* products have been inspected and their inspection information is then used to compute *x*_*j*+1_^**y**_*j*_^.

The decision as to whether to perform inspection on the *j* + 1th product or to stop the inspection procedure depends on their resulting “remaining expected cost" (REC), including the cost of inspection and the cost of shortage for the RUD. The optimal inspection policy is to choose the action that produces the smallest REC. The related notations for REC are defined as follows given that under the FPFI strategy where the RUD is *d* and the batch size is *N*, with the first *j* products whose inspection results are recorded being **y**_*j*_ and *N* − *j* being the uninspected products left:

 
*G*_*d*,*N*−*j*_^INSP^(*x*_*j*+1_^**y**_*j*_^): the REC if one chooses to inspect the *j* + 1th product and afterward an optimal inspection policy is followed.
 
*G*_*d*,*N*−*j*_^STOP^(*x*_*j*+1_^**y**_*j*_^): the REC if one chooses to terminate the inspection procedure at the *j* + 1th product; that is, only the first *j* products are inspected, and the rest of the products in the batch are discarded. In this case, the REC is equal to *sd*.
 
*G*_*d*,*N*−*j*_(*x*_*j*+1_^**y**_*j*_^): the minimal REC by performing an optimal inspection policy.

Note that

(4) $$\begin{array}{c} G_{d,N-j}\left(x_{j+1}^{y_{j}}\right)=\min\left\{G_{d,N-j}^{\text{INSP}}\left(x_{j+1}^{y_{j}}\right),G_{d,N-j}^{\text{STOP}}\left(x_{j+1}^{y_{j}}\right)=sd\right\}, \end{array}$$

where

(5) Gd,N−jINSP(xj+1yj)=γ+[θ0xj+1yj+θ1(1−xj+1yj)]×Gd−1,N−j−1(xj+2(yj,0))+[1−θ0xj+1yj−θ1(1−xj+1yj)]×Gd,N−j−1(xj+2(yj,1)).

A sufficient condition whereby the *j* + 1th product should be inspected is stated in the following lemma.

**Lemma**

If *x*_*j*+1_^**y**_*j*_^ > (*γ*/(*s* − *θ*_1_))/(*θ*_0_ − *θ*_1_), then the *j* + 1th product should be inspected.

Lemma 1 shows that if the probability that the *j* + 1th product is produced in the IN state is large enough, that is, larger than the threshold *x*_*j*+1_^**y**_*j*_^ > (*γ*/(*s* − *θ*_1_))/(*θ*_0_ − *θ*_1_), then inspecting the *j* + 1th product is attractive.

**Example**

**Figure 1:**
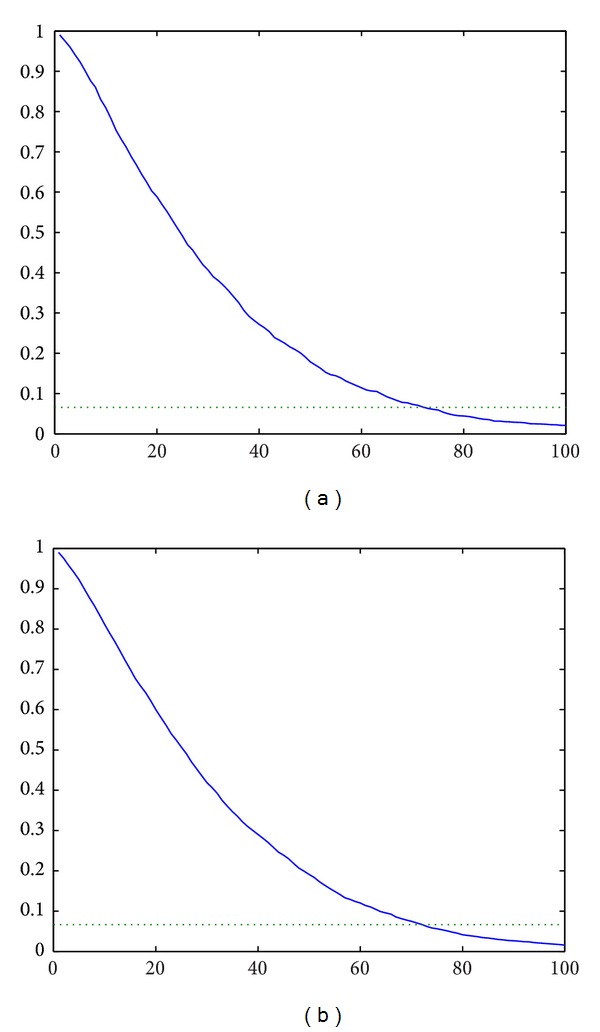
(a) The average value of *x*_*j*+1_^**y**_*j*_^ using 1200 simulations. (b) The average value of *x*_*j*+1_^**y**_*j*_^ using 3600 simulations.

To illustrate Lemma 1, the following parameter values are used: *θ*_0_ = 0.9, *θ*_1_ = 0.4, *γ* = 1.3, *s* = 3, *n*_*U*_ = 100 and ${\bar{P}}_{j}=q^{j^{\delta }}$ with *q* = 0.99 and *δ* = 1.3, which is the case when the process has a discrete Weibull shift distribution with an increasing hazard rate (see Nakagawa and Osaki [12]). If the probability that the *j* + 1th product is conforming is *θ*_0_ *x*_*j*+1_^**y**_*j*_^ + *θ*_1_(1 − *x*_*j*+1_^**y**_*j*_^) greater than *λ*, then the *j* + 1th product is classified as conforming, else the *j* + 1th product is regarded as nonconforming, where *λ* ∈ (0,1) is randomly generated. The average value of *x*_*j*+1_^**y**_*j*_^ with 1200 and 3600 simulations are drawn in Figures 1(a) and 1(b), respectively, where they indicate that *x*_*j*+1_^**y**_*j*_^ has a similar trend decreasing in *j* and the products produced before the 54th product should be inspected since the threshold is 0.0667.

Different from the case given in Lemma 1, one should always stop the inspection procedures if one of the following conditions occurs: (i) none of the uninspected products is left; then, the RUD is regarded as shortage; thus, *G*_*d*,0_(*x*_*N*+1_^**y**_*N*_^) = *sd* for *d* > 0; (ii) when the RUD is zero and the corresponding REC is zero; that is, *G*_0,*N*−*j*_(*x*_*j*+1_^**y**_*j*_^) = 0 for *j* = 0,1, 2,…, *N*.

Using (i) and (ii) in (5) for *j* = *N* − 1 gives *G*_*d*,1_^INSP^(*x*_*N*_^**y**_*N*−1_^) = *γ* + *sd* − *s*[*θ*_1_ + (*θ*_0_ − *θ*_1_)*x*_*N*_^**y**_*N*−1_^] which could be used as a boundary condition in computing RECs. That is,

(6) Gd,1(xNyN−1) =min⁡{Gd,1INSP(xNyN−1)=γ+sd−s[θ1+(θ0−θ1)xNyN−1],      Gd,1STOP(xNyN−1)=sd}.

As a summary, the following algorithm is proposed to find the optimal production batch size, *N*_*D*_, that minimizes the expected total cost, $\alpha +\beta N+G_{D,N}\left(x_{1}^{y_{0}}={\bar{P}}_{1}\right)$, by searching *N* = 1,2,…, *n*_*U*_.

**Step 1**

Input the model parameter value: *D*, *n*_*U*_, *θ*_0_, *θ*_1_, *α*, *β*, *γ*, *s*, and ${\bar{P}}_{j}$. Initially, set *d* = 0.

**Step 2**

Set *d* = *d* + 1 and *N* = 0.

**Step 3**

Set *N* = *N* + 1, and perform the following steps

 
*Step* **3.1*.* Sequentially compute *G*_*d*,*N*−*j*_(*x*_*j*+1_^**y**_*j*_^) from *j* = *N*, *N* − 1, *N* − 2,…, 0, where if *j* = *N*, then set *G*_*d*,0_(*x*_*N*+1_^**y**_*N*_^) = *sd*; else if *j* = *N* − 1, then compute *G*_*d*,1_(*x*_*N*+1_^**y**_*N*_^) through (6); otherwise, if *j* < *N* − 1, then compute *G*_*d*,*N*−*j*_(*x*_*j*+1_^**y**_*j*_^) through (4) and (5).
 
*Step* **3.2*.* If *d* ≤ *D* and *N* < *n*_*U*_, then go to Step 3; else if *d* < *D* and *N* = *n*_*U*_, then go to Step 2; otherwise, if *d* = *D* and *N* = *n*_*U*_, then go to Step 4.

**Step 4**

Output $N_{D}=\arg\min_{N=1,2,\ldots ,n_{U}}\left\{\alpha +\beta N+G_{D,N}\left(x_{1}^{y_{0}}={\bar{P}}_{1}\right)\right\}$ and STOP.

The computational complexity of performing the above proposed algorithm is significant with *O*(2^*n*_*U*_^ *n*_*U*_ *D*) for *D* < *n*_*U*_ and *O*(2^*n*_*U*_^ *n*_*U*_^2^) for *D* ≥ *n*_*U*_. One can use the following property provided by Anily and Grosfeld [10] to reduce the computations: *G*_*d*,*K*_(*x*_*j*_^**y**_*j*−1_^) = *G*_*K*,*K*_(*x*_*j*_^**y**_*j*−1_^) + *s*(*d* − *K*) for 0 ≤ *K* < *d*. That is, when *d* > *N* − *j*, Step 3.1 can be rewritten as *G*_*d*,*N*−*j*_(*x*_*j*+1_^**y**_*j*_^) = *G*_*N*−*j*,*N*−*j*_(*x*_*j*+1_^**y**_*j*_^) + *s*(*d* − *N* + *j*) for *j* < *N* − 1.

In Step 3, $G_{D,N}\left(x_{1}^{y_{0}}={\bar{P}}_{1}\right)$ can be theoretically obtained by applying the law of large numbers. That is, one can simulate the discussed PI problem a large number of times in order to compute their average total quality control cost as an approximation $G_{D,N}\left(x_{1}^{y_{0}}={\bar{P}}_{1}\right)$, where the simulation approach is the same as the one we have used in Example 2 in order to determine whether the *j*th product is conforming or not.

**Example 3**

**Figure 2:**
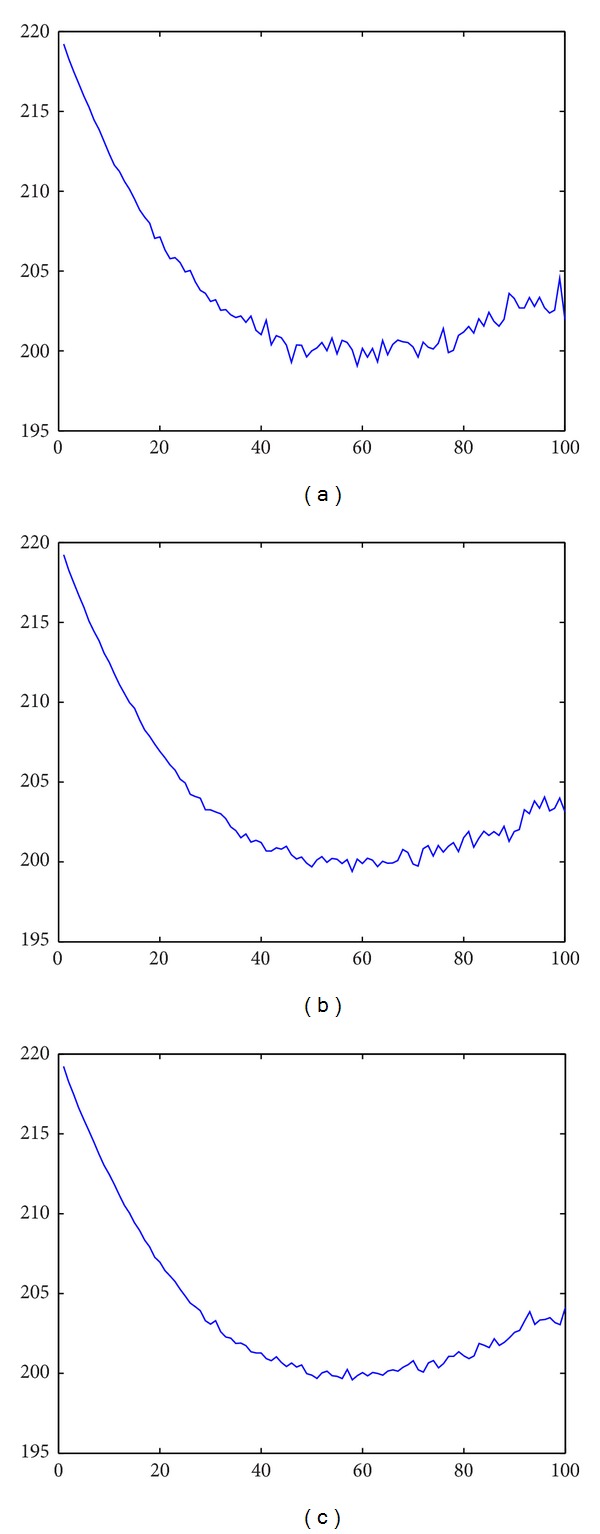
(a) The average total cost with different batch sizes (1200 simulations). (b) The average total cost with different batch sizes (3600 simulations). (c) The average total cost with different batch sizes (7200 simulations).

The same model parameter value and simulation approach used in Example 2 is then adopted to compute $G_{D,N}\left(x_{1}^{y_{0}}={\bar{P}}_{1}\right)$ with the extra parameter values: *α* = 10, *β* = 0.6, and *D* = 70. When we simulate the PI model 1200 times and the obtained average optimal lot size was 59, with the resulting average RUD being 7.5833, and the associated average total cost being 199.0653.  Figure 2(a) shows that the average total cost is not sensitive in terms of lot size around the optimal lot size 59.

Again, when 3600 simulations were performed, we obtained the average solution as follows: *N*_70_ = 58, the average RUD is 7.7803, and the average total cost is 199.4059.  Figure 2(b) depicts the average total cost with different lot sizes. When 7200 simulations were performed, we also have *N*_70_ = 58. The resulting average RUD is 7.8043 and the average total cost is 199.5970 (also see Figure 2(c)). This reveals that when the number of simulations becomes large enough, the average optimal PI solution converges. Note that in Figures 2(a)–2(c), the cost curve becomes much smoother when more simulations are performed.

**Figure 3:**
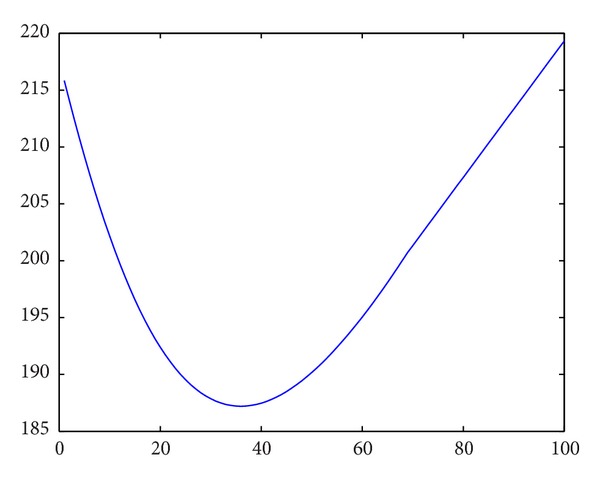
The expected total cost with different batch sizes.

It should be noticed that if only the last inspection information is used to infer the process quality of the next candidate product in terms of the inspection decision, the computational complexity dramatically reduces to *O*(2*n*_*U*_ *D*_0_) for *d* < *U* and *O*(2*n*_*U*_^2^) for *d* ≥ *U* when performing our proposed algorithm for an optimal PI policy, where *x*_*j*+1_^**y**_*j*_^ can be easily modified by replacing the index *i* = 1 with *i* = *j* − 1 in (1). Applying our proposed algorithm with the same parameter values given in Example 3, we obtained 31 as the optimal lot size and the corresponding total cost is 207.8792 (see Figure 3). Although the obtained optimal lot size 31 is an underestimate compared to the solution of Example 3, that is, when the simulation is run 7200 times, the percentage cost difference is only 4.15%, which is not that significant. This example shows that using a reduced amount of latest product quality information rather than all previous inspection information in order to determine the optimal PI policy may be a useful approach in some cases.

## 4. Conclusions 

This study extends Anily and Grosfeld's [10] production and inspection (PI) model with one with nonrigid demand to one that considers an arbitrary discrete probability distribution instead of being limited to the case where the process possesses a constant hazard rate. The extended mathematical model and an associated algorithm for the optimal PI policy are established. Our proposed PI model should be able to be applied more widely in industry than the previous approach.

The incorporation of an inspection error into the PI model will need further research (see Grosfeld-Nir et al. [13]). When product inspection has a type I error, that is, a conforming product is inspected to be nonconforming, it does not only increase the inspection cost but also the production cost. In extremity, if the probability of the type I inspection error is one, then one will never obtain a conforming product through inspection. On the other hand, when type II inspection errors are present, that is, a nonconforming product is inspected to be conforming, it is impossible to achieve the zero defect condition, and thus, the impact of a warranty policy, such as a free repair warranty policy, on the PI problem needs to be included in order to determine its effect on the optimal PI policy.

## Appendix

*A derivation of x*_*j*+1_^**y**_*j*_^. First notice that *x*_*j*+1_^**y**_*j*_^ = Pr(*M* > *j* + 1 | **Y**_*j*_ = **y**_*j*_). Furthermore, it is easy to see that

(A.1)Pr(M>j+1 ∣ Yj=yj)  =Pr(M>j+1,M>j ∣ Yj=yj)  =Pr(M>j+1,M>j,Yj=yj)Pr(Yj=yj)  =Pr(M>j+1,M>j,Yj=yj)Pr(M>j,Yj=yj)   ·Pr(M>j,Yj=yj)Pr(Yj=yj)  =Pr(M>j+1 ∣ M>j,Yj=yj)   ×Pr(M>j ∣ Yj=yj).

The last equation can be rewritten as.

$$\begin{array}{cc} \left(\text{A}.2\right) & \text{Pr}\left(M>j+1\;|\;M>j\right)\text{Pr}\left(M>j\;|\;Y_{j}=y_{j}\right). \end{array}$$

This is because when given that the *j*th product is produced in the IN state, the status of the production system while producing the *j* + 1th product is independent of the products quality status of the previous *j* products, **Y**_*j*_ (see Anily and Grosfeld [10]). Consequently, $\left (\text {A}.2\right )$ can be easily expressed as (1), where $\text{Pr}\left(M>j+1|M>j\right)={\bar{P}}_{j+1}/{\bar{P}}_{j}$ and

(A.3)Pr(M>j ∣ Yj=yj) =Pr(Yj=yj ∣ M>j)Pr(M>j)  ×(∑k=1j{Pr(Yj=yj ∣ M=k)Pr(M=k)}     +Pr(Yj=yj ∣ M>j)Pr(M>j))−1 =∏i=1j[(1−yi)θ0+yi(1−θ0)]P¯j  ×(∑i=1j{∏k=1i−1[(1−yk)θ0+yk(1−θ0)]        ×∏k=ij[(1−yk)θ1+yk(1−θ1)]}pi      +∏i=1j[(1−yi)θ0+yi(1−θ0)]P¯j)

are used.

Proof of Lemma 1
If *x*_*j*+1_^**y**_*j*_^ > (*γ*/(*s* − *θ*_1_))/(*θ*_0_ − *θ*_1_), then it is easy to see that *s*[*θ*_1_ + (*θ*_0_ − *θ*_1_)*x*_*j*+1_^**y**_*j*_^] > *γ*. This implies that

$$\begin{array}{cc} \left(\text{A}.4\right) & G_{D,N-j}^{\text{STOP}}\left(x_{j+1}^{y_{j}}\right)=sD>\gamma +sD-s\left[\theta_{1}+\left(\theta_{0}-\theta_{1}\right)x_{j+1}^{y_{j}}\right]. \end{array}$$

In addition, from (4) and (5), it is easy to see that

(A.5)GD,N−jINSP(xj+1yj)≤γ+[θ1+(θ0−θ1)xj+1yj]s(D−1)+[1−θ1−(θ0−θ1)xj+1yj]sD.

Or, equivalently,

$$\begin{array}{cc} \left(\text{A}.6\right) & G_{D,N-j}^{\text{INSP}}\left(x_{j+1}^{y_{j}}\right)\leq \gamma +sD-s\left[\theta_{1}+\left(\theta_{0}-\theta_{1}\right)x_{j+1}^{y_{j}}\right]. \end{array}$$

Therefore, when *x*_*j*+1_^**y**_*j*_^ > (*γ*/(*s* − *θ*_1_))/(*θ*_0_ − *θ*_1_), one has

(A.7)GD,N−jSTOP(xj+1yj)=sD>γ+sD−s[θ1+(θ0−θ1)xj+1yj]≥GD,N−jINSP(xj+1yj).
